# Fractal Dimension Characterization of Joint Surface Morphology on Dissimilar Friction Stir Lap Welding of Al/Mg

**DOI:** 10.3390/ma12233941

**Published:** 2019-11-28

**Authors:** Yadong Zhao, Yalong Luo, Zhipeng Zhang, Haixiao Zhang, Xuefeng Guo, Shuguang Wang, Hongbao Cui, Yangming Zhang

**Affiliations:** 1 School of Materials Science and Engineering, Henan Polytechnic University, Jiaozuo 454000, Chinacuihongbao@hpu.edu.cn (H.C.); 2 School of Mechanical Engineering, Anyang Institute of Technology, Anyang 455000, Chinaaygxywsg@163.com (S.W.);

**Keywords:** friction stir lap welding (FSLW), fractal dimension, response surface method

## Abstract

The joint surface of the 1060 aluminum and AZ31 magnesium alloy was prepared through friction stir lap welding (FSLW) under different welding process parameters. The joint surface was characterized three-dimensionally using a three-dimensional (3D) optical profiler, and the coordinate data were obtained. The fractal dimension of the joint surface was calculated by the box-width transformation method using a MATLAB program. Furthermore, the influence of the welding process parameters on the fractal dimension of the joint surface was studied. The response surface model was established based on the principle of central composite design (CCD), and analysis of variance (ANOVA) was carried out to test the accuracy of the response surface. The results showed that the joint surface morphology had fractal characteristics, and the fractal dimension could be used as an index to characterize the quality of the joint surface. The change of the welding process parameters had a great impact on the fractal dimension of the joint surface, the interaction between the parameters was small, and the fitting accuracy of the response surface model was high. The fractal dimension of the joint surface decreased with the increase in the welding and rotational speeds and the effect of the rotational speed was more significant.

## 1. Introduction

Aluminum alloys have good plasticity and high corrosion resistance. Magnesium alloys have low densities and high specific strengths and heat transfer capacities. Magnesium and aluminum alloys are lightweight materials widely used in aerospace, automobiles, ships, and other industrial fields [1,2]. Due to the differences in the physical and chemical properties of these two kinds of lightweight alloy materials, welding defects, such as cracks, porosity, and inclusions, are easily generated during the welding process, resulting in low strengths of the welded joints and even ineffective connections [3]. Friction stir welding (FSW) is a new type of solid-phase connection technology, which is mainly realized by the plastic flow of high-temperature metals. It has become an ideal connection technology for these two lightweight alloy materials because it produces no smoke or arc light, involves a simple pre-welding treatment, and produces small welding deformation during the welding process. Furthermore, it prevents defects such as cracks, porosity, and inclusions caused by fusion welding [4].

The forming quality of the joint surface of FSW directly affects the performance of the welded joint. In recent years, researchers have mainly relied on the naked eye to judge the forming quality of the FSW, tungsten inter gas welding, laser welding joint surface and coatings [5,6,7,8,9,10,11,12,13,14], which has certain limitations. Liu et al. [5] used FSW to conduct the butt-welding process of the AZ91 magnesium alloy and A383 aluminum alloy. After welding, the forming quality of the joint surface was investigated when the welding was 40 mm/min and the rotational speeds were 700, 900, 1100, 1300, and 1500 rpm. The qualitative judgment of the forming quality of the joint surface was made using a camera and the naked eye. The best forming quality of the joint surface was obtained when the welding speed was 40 mm/min and the rotational speed was 900 rpm. Similarly, Mehta et al. [6], Zhang et al. [7], and Abdollahzadeh et al. [8] studied the friction stir butt welding of the AA6061 aluminum alloy, AM60 aluminum alloy, 6061-T6 aluminum alloy, and AZ31 magnesium alloy, which are dissimilar materials, and visual observations were used to make qualitative judgments of the forming quality of the joint surface. To observe the forming quality of the joint surface more intuitively, researchers have used a 3D optical profiler to characterize it. Boccarusso et al. [15] studied the dissimilar friction stir lap welding (FSLW) of the AA6082 aluminum alloy and AZ31 magnesium alloy. After welding, the joint surface was observed using a 3D optical profiler. Patel et al. [16] also carried out 3D characterizations on the joint surface of a magnesium alloy after FSW. They used the surface roughness of the cross-section of the joint at a certain position in the longitudinal and transverse directions to represent the forming quality of the joint surface. This method only partially represented the forming quality of the joint surface, and it was limited without additional quantitative analysis of the forming quality of the whole surface.

Aluminum and magnesium alloys were examined in this study. Joints under different welding process parameters were constructed, and the forming qualities of the FSW joint surfaces of the dissimilar aluminum and magnesium metals were investigated in detail using a 3D optical profiler combined with a MATLAB self-compiled program. A new method to judge the forming quality of the joint surface was proposed, which used the fractal dimension D to characterize it. The response surface method was used to study the influence of different welding process parameters on the fractal dimension of the joint surface. The results in this paper lay a theoretical foundation and have practical significance for expanding the application of FSW to connect aluminum and magnesium alloys.

## 2. Materials and Methods

The dimensions of the 1060 aluminum alloy specimens were 200 mm × 150 mm × 2 mm, and those of the AZ31B magnesium alloy specimens were 200 mm × 150 mm × 3 mm. The AZ31B magnesium alloy had the following composition (in wt%): Al: 3.1, Mn: 0.48, Zn: 0.88, Si: 0.11, Fe: 0.0027, Cu: 0.0015, Ni: 0.0005, Mg: Balance. The 1060 aluminum alloy had the following composition (in wt%): Al: 99.6, the total of Cu, Mn and Si was less than 0.4. The HT-JM16 × 8/1 gantry-type one-dimensional FSW equipment was used. The work table size of FSW equipment was 1000 mm in length and 800 mm in width. The spindle maximum speed was 2500 rpm. *X* axis and *Y* axis and *Z* axis travel was 800 mm, 600 mm, and 300 mm, respectively. The lap joint welding method was adopted, with an aluminum plate on the top and a magnesium plate on the bottom. The length of lap joint area was 45 mm. Before welding, the surfaces of the plates in the overlapping contact area of the magnesium and aluminum were sanded and wiped clean with alcohol, after which they were overlapped on the workbench and fixed with clamps. During welding, a mixing head with a shaft shoulder diameter of 14 mm, a mixing needle length of 4 mm, and a bottom diameter of 3 mm was used. The tool shoulder plunge depth was 0.2 mm, and the tool tilt angle was 2.5°.

When the rotational speeds were 1000, 1500, and 2000 rpm, the welding speeds were 30, 50, and 70 mm/min, respectively. Nine groups of different joint surfaces of the FSLW without evident defects were obtained. A NANOVEA ST400 3D optical profiler (NANOVEA company, USA) was used to scan the joint surface. Before scanning, the surface was washed with alcohol, and then it was placed on the workbench and fixed to prevent it from moving. Finally, a MATLAB 2018a program was run that used the 3D coordinates of the scanned surface to calculate the fractal dimension of the joint surface.

The response surface methodology (RSM) [17] is an empirical statistical modeling technology that uses trial data to solve multiple equations and carry out multiple regression analysis. It allows the statistical analysis of different factors and variables. Based on the central composite design (CCD) [18], with the rotational and welding speeds as two dependent variables and the fractal dimension D as the response surface value, a quadratic polynomial was used to fit the functional relationship between the designed parameters and the response surface value. The least squares method was used to obtain the undetermined coefficients of each item, after which the function model of the response surface was fitted. Finally, ANOVA and an accuracy test of the model were carried out.

## 3. Results and Analysis

### 3.1. 3D Joint Surface Morphology

**Figure 1:**
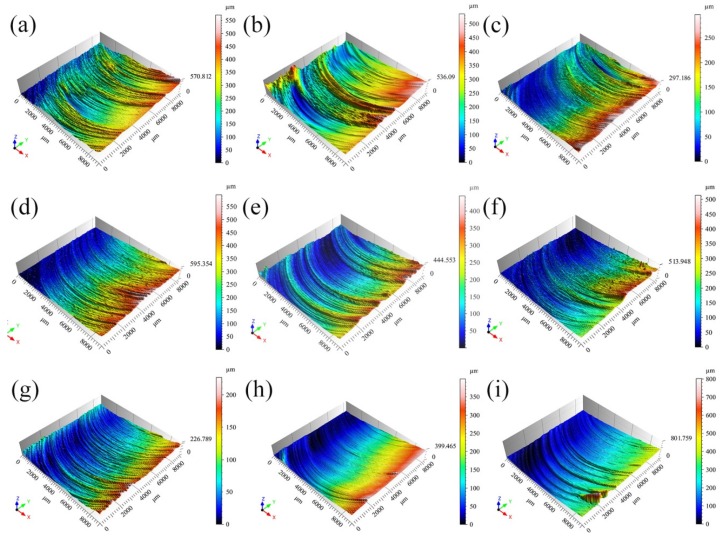
Friction stir welding (FSW) 3D joint surface morphology: (**a**) 1000 rpm, 30 mm/min, (**b**) 1000 rpm, 50 mm/min, (**c**) 1000 rpm, 70 mm/min, (**d**) 1500 rpm, 30 mm/min, (**e**) 1500 rpm, 50 mm/min, (**f**) 1500 rpm, 70 mm/min, (**g**) 2000 rpm, 30 mm/min, (**h**) 2000 rpm, 50 mm/min, and (**i**) 2000 rpm, 70 mm/min.

**Figure 2:**
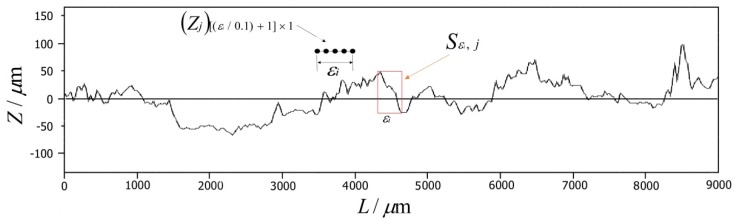
Calculation of the fractal dimension of a cross-sectional curve of the joint surface.

Figure 1 shows the 3D joint surface morphology of the FSLW of the aluminum and magnesium metals for different welding process parameters. In Figure 1a, the black arrow indicates the welding direction. The mixing head rotated counterclockwise, and the *Z* axis represents the height of the joint surface. Red and yellow areas indicate a low surface finish, whereas blue areas indicate a high surface finish. The color distributions of the 3D morphologies for different welding process parameters had similar characteristics—the colors of the front side of the joint surface were mainly red and yellow, and the color of the back side was mainly blue, indicating that the surface finish of the back side of the weld was higher than that of the front side (Figure 1). On the cross-sectional curve at the center of the weld along the *Y* direction (Figure 2), the peak and valley values of all the joint surface heights were within the range of 50–300 µm. As shown in Figure 1, when the rotational speed was fixed, the size of the blue area on the joint surface gradually increased with the increase in the welding speed. When the welding speed was fixed, the sizes of the red and yellow areas on the joint surface gradually decreased with the increase in the rotational speed. These results show that the surface finish of the weld increased with the increase in the rotational and welding speeds.

### 3.2. Calculation of Fractal Dimension of Cross-Sectional Curve of Joint Surface

As a measure of the irregularity of a complex shape, the fractal dimension reflects the effectiveness of the space occupied by the complex shape. Box-counting N(ε) was refer to the number obtained by covering the measured form with basic units. In the present work, using a scale ε measured N(ε) then the fractal dimension D can be calculated as follows:

(1) $$D=\underset{\varepsilon \to 0}{lim}\frac{\ln N\left(\varepsilon \right)}{\ln\left(\frac{1}{\varepsilon }\right)}$$

Using the coordinate data of the 3D joint surface morphology obtained by the scanning sampling, the cross-sectional curves at different positions in the welding direction and the weld width direction could be obtained. The box-width transformation method [19,20] was used to calculate the fractal dimension of the curve. The calculation process is as follows.

The fractal curve was covered by a rectangle with a width of ε, as shown in Figure 2. The height of the rectangle was determined by the difference between the coordinates in the thickness direction of the highest and lowest points of the fractal curve in the rectangle frame. The rectangle frame was moved step by step to completely cover the fractal curve, and the area of each frame was added to obtain the total area, *S_ε_*. By changing the width of the rectangle to *ε_i_* systematically, the corresponding total area, *S_εi_*, could be obtained. The total area, *S_εi_*, was divided by *ε_i_*^2^ to obtain the box count:

(2) $$N(\varepsilon_{i})=S_{\varepsilon_{i}}/\varepsilon_{i}^{2}=\sum_{j=1}^{L/\varepsilon_{i}}S_{\varepsilon ,j}/\varepsilon_{i}^{2}=\sum_{j=1}^{L/\varepsilon_{i}}(\max(Z_{j}{)}_{[(\varepsilon_{i}/0.1)+1]\times 1}-\min{\left(Z_{j}\right)}_{[(\varepsilon_{i}/0.1)+1]\times 1})/\varepsilon_{i}$$

where *L* is the cross-sectional width, *S*_*ε*,*j*_ is the area of the *j*-th rectangular frame with a width of *ε_i_*, *Z_j_* is a [(*ε_i_*/0.1) + 1] × 1 matrix formed by the coordinate value in the thickness direction with (*ε_i_*/0.1) + 1 scanning points covered by the *j*-th rectangular frame, and 0.1 is the scanning step.

According to Equation (2), a smaller width and height of the rectangle corresponds to a greater total number of rectangles required to cover the curve and a greater corresponding box count. However, regardless of the measurement method, it is impossible to achieve a width, ε, that tends to zero. Therefore, the measurement scale can only be continuously reduced to achieve a certain accuracy based on the measurement technology. In this study, the minimum width, ε, was taken as the scanning step length. Based on the obtained morphology, this value was far smaller than the joint size, so it met the accuracy requirements.

**Figure 3:**
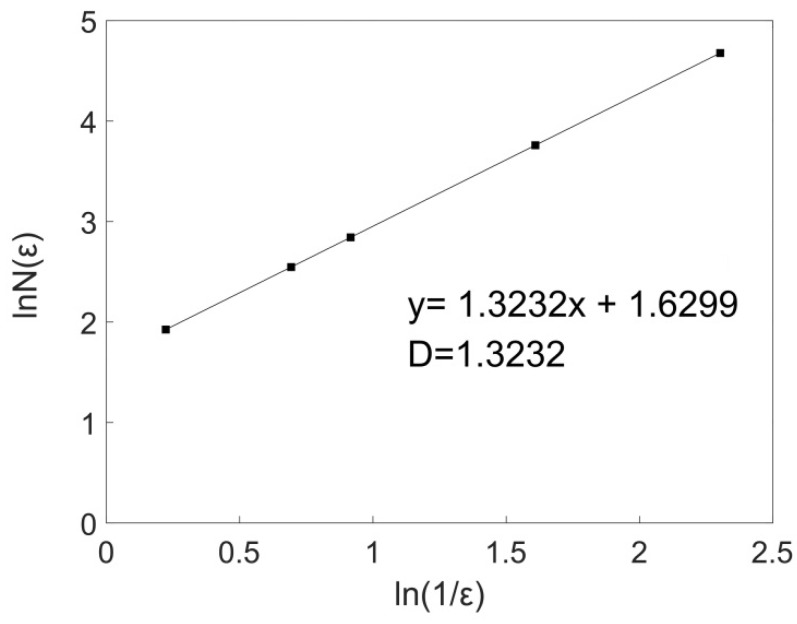
Double logarithmic curve of the box count and box width of the cross-sectional curve of the joint surface when *x* = 4 mm.

Under a rotational speed of 1500 rpm and welding speed of 50 mm/min, the fractal dimension (D_C_) of 10 curves collected by 10 equal divisions in the direction of welding and the direction of the weld width were calculated using a MATLAB program. The average fractal dimension of 10 curves was taken as the fractal dimension of the joint surface in this welding direction. Taking the cross-sectional curve with a width of *x* = 4 mm as an example, the distribution curve of $\ln N(\varepsilon_{i})\sim \ln(1/\varepsilon_{i})$ was plotted, and the slope of the linear region was used as the fractal dimension of the fractal curve. Figure 3 shows the double logarithm relationship between the box count and the box width of the cross-sectional curve with a width of *x* = 4 mm on the welding surface. There was a very strong linear correlation between $\ln N(\varepsilon_{i})$ and $\ln(1/\varepsilon_{i})$, indicating that the curve had fractal characteristics (See Figure 3). Table 1 lists the calculated fractal dimension of the 10 curves in the welding direction under the welding process parameters for a rotational speed of 1500 rpm and welding speed of 50 mm/min, and the fractal dimension (D_C_) in this direction was 1.3141.

**Figure 4:**
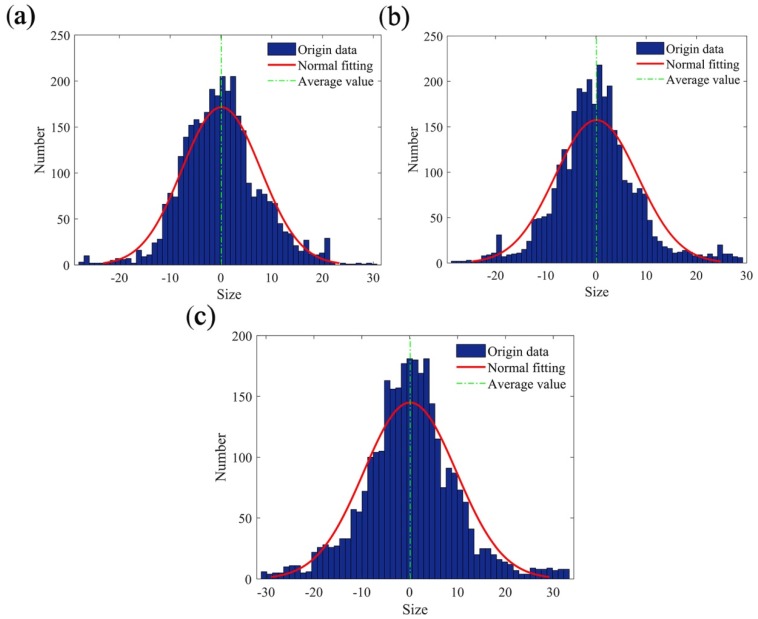
Normal distribution fit of the cross-sectional curve of the joint surface at different positions: (**a**) *x* = 1 mm, (**b**) *x* = 4 mm, and (**c**) *x* = 7 mm.

In this paper, the curves at *x* = 1, 4, and 7 mm were randomly selected for fitting, and the curves at the three positions basically followed a normal distribution (Figure 4):

(3) $$F(x)=\frac{1}{\sqrt{2\pi }\sigma }e^{-\frac{{\left(x-\mu \right)}^{2}}{2\sigma^{2}}}$$

where μ and σ represent the average value and standard deviation, respectively.

As shown in Table 1, when *x* = 1 mm, the fractal dimension of the curve was 1.3313, when *x* = 4 mm, the fractal dimension of curve was 1.3232, and when *x* = 7 mm, the fractal dimension of the curve was 1.3152. The fractal dimensions of the curves at the three positions were very similar. Figure 4 and Table 2 show that when *x* = 1 mm, μ was 0.0744 and σ was 3.692; when *x* = 4 mm, μ was 0.0882 and σ was 3.808; when *x* = 7 mm, μ was 0.0945 and σ was 3.976. The average value, μ, and the standard deviation, σ, of the three positions were very similar, which is consistent with the results in Table 1. This verified that the calculation process of the fractal dimension of the cross-sectional curve of the joint surface was correct.

### 3.3. Calculation of Fractal Dimension of Curved Surface of Joint Surface

**Figure 5:**
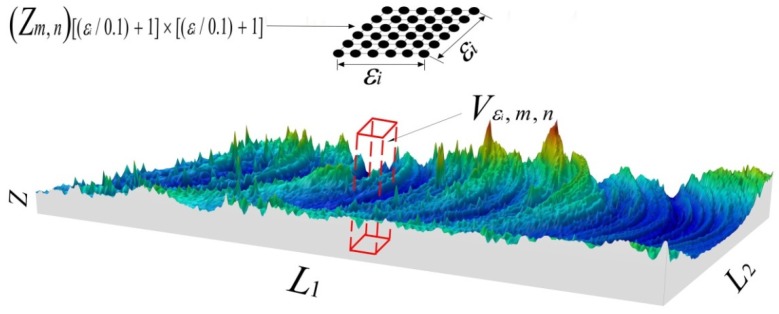
Calculation diagram of the fractal dimension of the joint surface.

The method of using the fractal dimension to quantitatively characterize the 3D morphology was similar to that of characterizing the fractal dimension of the cross-sectional curve of the joint surface. A rectangle frame with a width of ε was replaced by a square column with a bottom surface of ε × ε (Figure 5). The height of the square column was determined by the difference between the coordinates in the thickness direction of the highest and lowest points of the scanning surface within the range of ε × ε, and the square column was moved step by step to completely cover the curved surface of the joint. The volumes of the square columns were added to obtain the total volume, *V_ε_*. By systematically changing the width of the bottom of the square column to *ε_i_*, the corresponding total volume, *V_ε_*, could be obtained. The total volume *V_ε_* was divided by *ε_i_* to obtain the box count:

(4) $$\begin{array}{l} N(\varepsilon_{i})=V\varepsilon_{i}/{\varepsilon_{i}}^{3}=\sum_{m=1}^{L1/\varepsilon_{i}}\sum_{n=1}^{L2/\varepsilon_{i}}V\varepsilon_{i},m,n/{\varepsilon_{i}}^{3}\\ =\sum_{m=1}^{L1/\varepsilon_{i}}\sum_{n=1}^{L2/\varepsilon_{i}}\left(\max(\max(Z_{m,n})[\varepsilon_{i}/0.1+1]\times [\varepsilon_{i}/0.1+1])-\min(\min(Z_{m,n})[\varepsilon_{i}/0.1+1]\times [\varepsilon_{i}/0.1+1])\right)/{\varepsilon_{i}}^{3} \end{array}$$

where *L*_1_ and *L*_2_ are the scanning width in the direction of the welding speed and welding width, respectively, *V*_*εi*,*m*,*n*_ is the volume of the square column at the position of (*m*,*n*) with a bottom of *ε_i_* × *ε_i_*. *Z_m,n_* is the [(*ε_i_*/0.1) + 1] × [(*ε_i_*/0.1) + 1] order matrix formed by the coordinate values in the thickness direction of the scanning points within the coverage of this square column, and 0.1 is the scanning step length.

**Figure 6:**
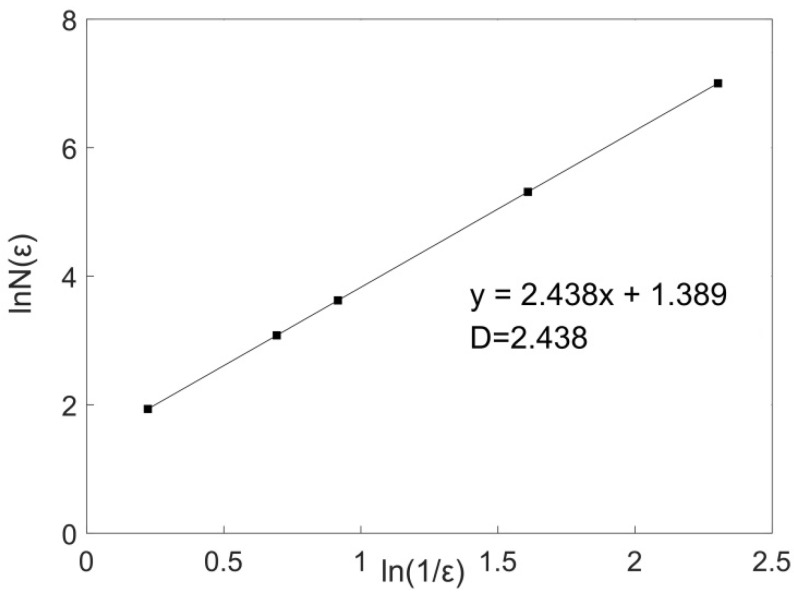
Double logarithmic curve of the box count and box width of the joint surface.

Under a rotational speed of 1500 rpm and welding speed of 50 mm/min, the fractal dimension of the joint surface (D_A_) was calculated. The above process was conducted in MATLAB, and the $\ln N(\varepsilon_{i})\sim \ln(1/\varepsilon_{i})$ distribution curve was plotted. The slope of the linear part of the curve was taken as the fractal dimension of the joint surface. The calculated results are listed in Table 3. Figure 6 shows the double logarithm relationship between box count and box width under the welding process parameters. There was a very high linear correlation between $\ln N(\varepsilon_{i})$ and $\ln(1/\varepsilon_{i})$, indicating that the surface morphology had fractal characteristics, and the fractal dimension D_A_ was 2.438. This calculation result was correct and satisfied the relationship between D_A_ and D_C_, which was D_A_ ≈ D_C_ + 1.

### 3.4. Influence of Welding Process Parameters on Fractal Dimension

To study the influence of the rotational and welding speeds on the fractal dimension of the joint surface, the rotational speed was set to 1000, 1500, and 2000 rpm, and the welding speed was set to 30, 50, and 70 mm/min. The fractal dimension D_C_ was used as the response surface value. The CCD method was adopted, and certain data were obtained from experiments. A multiple quadratic regression equation was used to fit the functional relationship between the factors and response value. The coding of the factor level is shown in Table 4. The central composite model in the Design Expert software was used to build the response surface. The CCD of the response surface was obtained by two factor variables. The minimum and maximum coding values of the two factor variables were −1 and 1, respectively, and the middle value was 0. The coding factor level is shown in Table 4, and the designed matrix and test results are shown in Table 5.

According to Table 5 and Table 6, the independent variables affecting the response surface value of the fractal dimension in the test were the rotational speed **ω** and welding speed **ν**. For the two variables, the expression of the quadratic linear regression equation “*y*” was as follows [21]:

(5) $$y=b_{0}+\sum b_{i}x_{i}+\sum b_{ij}x_{i}x_{j}+\sum b_{ii}x_{i}^{2}$$

where *b*_0_ is the intercept term, *b_i_* is the linear term, *b_ij_* is the interaction term, and *x_i_* and *x_j_* are designed parameters. In this paper, the response value was the fractal dimension D, which was a function of the rotational speed **ω** and the welding speed **ν**. Equation (5) can be expressed as follows:

(6) $$D=b_{0}+b_{1}\omega +b_{2}v+b_{12}\omega v+b_{11}\omega^{2}+b_{22}v^{2}$$

The rotational speed **ω** and welding speed **ν** of the designed parameters were selected as the *X* and *Y* coordinate axes, respectively. The following second-order polynomial equation was obtained through multiple regression analysis of the designed matrix and response values in Table 6:

(7) $$\begin{array}{cc} D= & 1.37529-1011308\times 10^{-3}A+1.26398\times 10^{-5}B+3.6985\times 10^{-7}AB\\ & -4.48966\times 10^{-6}A^{2}-2.03108\times 10^{-8}B^{2} \end{array}$$

**Figure 7:**
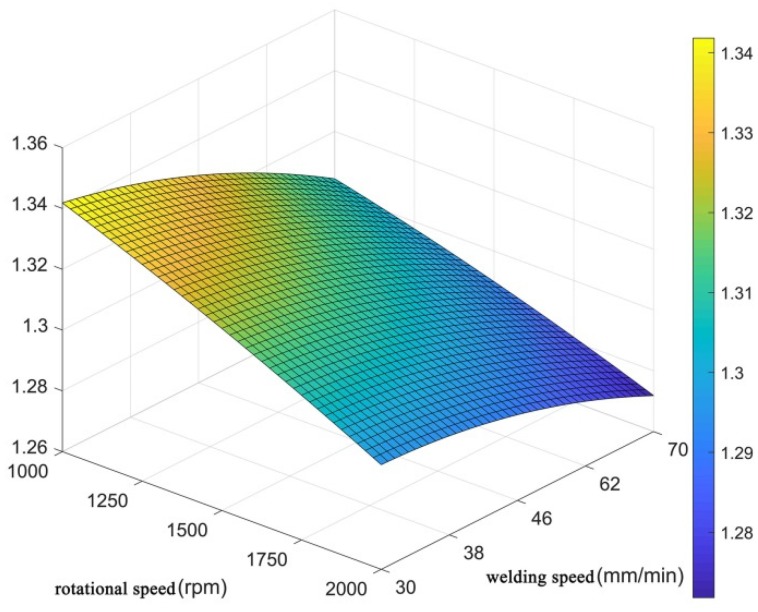
Response surface of the welding process parameters to the fractal dimension.

To better observe the relationship between the welding process parameters and fractal dimension, Equation (7) was fitted to obtain a 3D response surface, as shown in Figure 7. The response surface could directly reflect the relationship between the welding process parameters and the fractal dimension, and the influence of the welding process parameters on the fractal dimension D_C_ could thus be obtained. As shown in Figure 7, when the rotational speed was fixed, the fractal dimension D_C_ decreased with the increase in the welding speed; when the welding speed was fixed, the fractal dimension D_C_ decreased with the increase in the rotation speed.

The accuracy of the response surface is the basis for ensuring the effectiveness of the test design and response surface function, and it is necessary for conducting further analysis using the model. The accuracy of the response surface function and the significance of the selected designed parameters can be obtained through an accuracy test of the response surface, which is useful for judging whether the selected designed parameters are reasonable. The response surface model was analyzed using ANONA in the Design Expert software, and the results are shown in Table 6. In Table 6, the *F* value and probability *P* represent the significance of the correlation coefficient. A larger *F* value and smaller *P* value corresponded to a more significant correlation coefficient. Table 6 shows that the fitting accuracy of the model was high, and the designed parameters, ω and ν, were very significant, which indicated that the selection of the designed process parameters was reasonable and could reflect the change of the fractal dimension. The interaction between ω and ν was of low significance, indicating that the designed parameters had little correlation. The *F* value of ω was 364.86, which was higher than that of ν (198.09). The result indicated that the effect on the fractal dimension D of the rotational speed was more significant than the welding speed. The closer the coefficient of determination (*R*^2^) of the response surface function was to 1, the higher the fitting degree [14]. The coefficient of determination in this study was 0.9881, indicating that the fitting degree of response surface function was high.

**Figure 8:**
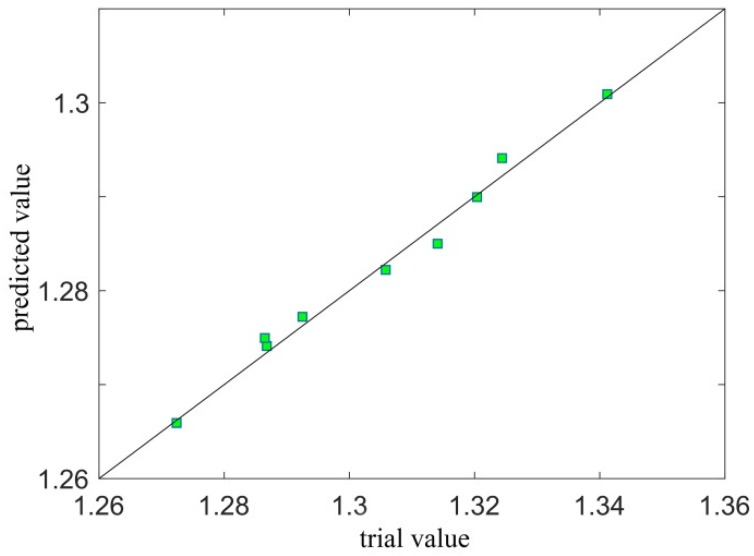
Correlation between the measured and predicted values.

**Figure 9:**
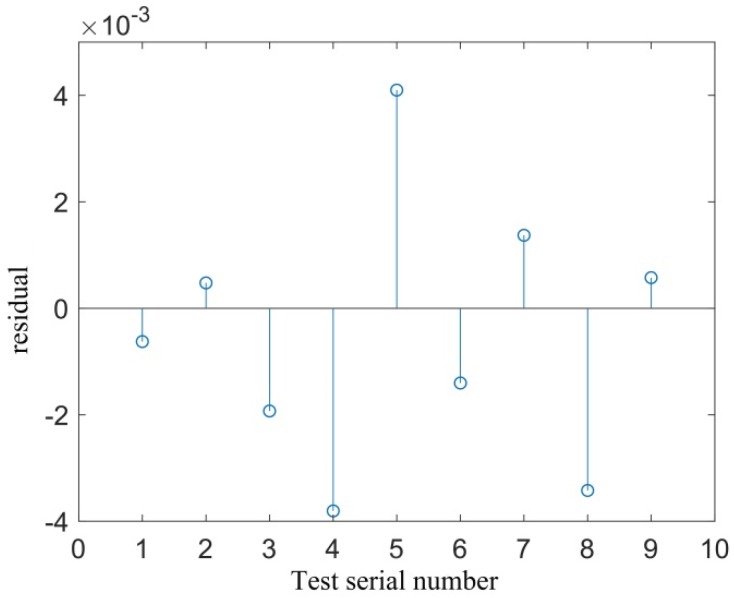
Residual diagram of the measured and predicted values.

To better observe the fitting accuracy of response surface, the correlation diagram and residual distribution diagram of the experimental and predicted values are given in Figure 8 and Figure 9, respectively. Figure 8 shows that all fractal dimensions D were near the 45° diagonal, and the residual value was basically within the range of 0.005 (Figure 9), indicating that the predicted value of the fitted response surface function was quite close to the actual value.

## 4. Conclusions

The joint surface of the dissimilar metals, aluminum and magnesium, was constructed by FSLW. The joint surface was scanned by a 3D non-contact optical profiler, and the 3D surface morphology data under different welding process parameters were obtained. The fractal dimensions of the cross-sectional curve and curved surface of the joint surface were calculated using the box-width transformation method and MATLAB programming. The response surface maps of the rotational speed, welding speed, and fractal dimension were obtained based on the CCD method. The accuracy of the response surface function model was tested, and the following conclusions were obtained:

1. The box-width transformation method was used to calculate the fractal dimensions of the cross-sectional curve and the curved surface of the joint surface, and it was found that there was a very high linear correlation between $\ln N(\varepsilon_{i})$ and $\ln(1/\varepsilon_{i})$. This showed that its morphology had fractal characteristics, and the fractal dimension D could be used as an indicator of the quality of the joint surface.
2. A high-accuracy response surface model that related the welding process parameters and fractal dimension was obtained using the CCD method, which directly reflected the influence of the process parameters on the fractal dimension. When the rotational speed was fixed, the fractal dimension D of the joint surface decreased with the increase in the welding speed. When the welding speed was fixed, the fractal dimension D decreased with the increase in the rotational speed. The effect of the rotational speed was more significant.

## Figures and Tables

**Table 1:** Fractal dimension of cross-sectional curve in 10 equal divisions of joint surface under a rotational speed of 1500 rpm and welding speed of 50 mm/min.

Calculated Curve No.	Section Position (mm)	Box Count					Curvilinear Fractal Dimension D
		*N(0.8)*	*N(0.5)*	*N(0.4)*	*N(0.2)*	*N(0.1)*	
1	*x* = 0	5.2	9.3	12.4	29.8	71.7	1.2658
2	*x* = 1	6.5	12.1	16.3	41.1	103.4	1.3313
3	*x* = 2	6.4	12	16.1	40.3	101.1	1.3256
4	*x* = 3	6.9	12.9	17.4	43.9	110.9	1.3382
5	*x* = 4	6.9	12.8	17.2	42.9	107.4	1.3232
6	*x* = 5	6.4	11.9	16	40.2	100.9	1.3278
7	*x* = 6	6.2	11	14.5	34	79.8	1.2992
8	*x* = 7	6.4	11.9	15.9	39.7	98.9	1.3152
9	*x* = 8	6.6	12.3	16.5	41.2	102.8	1.3183
10	*x* = 9	6.3	11.6	15.5	38.1	93.7	1.2964
Average value							1.3141

**Table 2:** Fitting parameters of the normal distribution of the cross-sectional curve of joint surface.

Section Position	Average Value μ	Standard Deviation σ
*x* = 1 mm	0.0744	3.692
*x* = 4 mm	0.0882	3.808
*x* = 7 mm	0.0945	3.976

**Table 3:** Calculation of fractal dimension of a curve on the joint surface.

*ε_i_*	N(*ε_i_*)	Surface Fractal Dimension D
0.8	6.9	
0.5	21.7	
0.4	37.4	2.438
0.2	202.9	
0.1	1099.6	

**Table 4:** Independent variable and coding level of the central composite design (CCD).

Symbol	Factor	Level Coding		
		−1	0	1
A	Rotational speed (rpm)	1000	1500	2000
B	Welding speed (mm/min)	30	50	70

**Table 5:** Designed matrix and test result.

Serial Number	Welding Speed (mm/min)	Rotational Speed (rpm)	Fractal Dimension
1	30	1000	1.3412
2	50	1000	1.3204
3	70	1000	1.2925
4	30	1500	1.3244
5	50	1500	1.3141
6	70	1500	1.2868
7	30	2000	1.3058
8	50	2000	1.2865
9	70	2000	1.2724

**Table 6:** ANOVA of the response surface function.

Source	Sum of Squares	df	Mean Square	*F* Value	*p*-Value Prob > *F*	
Model	0.005204	5	0.001041	116.07	<0.0001	significant
ω	0.003271	1	0.003271	364.86	<0.0001	
ν	0.001776	1	0.001776	198.09	<0.0001	
ων	0.0000758	1	0.0000758	8.45	0.0227	
ω^2^	0.000008844	1	0.000008844	0.99	0.3537	
ν^2^	0.000067	1	0.000067	7.47	0.0292	
Residual	0.00006276	7	0.000008966			
Lack of Fit	0.00006276	3	0.00002092			
Pure Error	0	4	0			
Cor Total	0.005266	12				
